# The Nutritional Value of Biowaste Bovine Slaughterhouse Meals for Monogastric Species Feeding: The Guinea Pig as an Animal Model

**DOI:** 10.3390/ani14071129

**Published:** 2024-04-08

**Authors:** Doris Chirinos-Peinado, Jorge Castro-Bedriñana, Patricia Álvaro-Ordoñez, Rolando Quispe-Ramos, Edgar García-Olarte, Elva Ríos-Ríos

**Affiliations:** 1 Nutritional Food Safety Research Center, Faculty of Zootechnics, Universidad Nacional del Centro del Perú, Huancayo 12000, Peru; dchirinos@uncp.edu.pe; 2 Specialized Institute, Faculty of Zootechnics, Universidad Nacional del Centro del Perú, Huancayo 12000, Peru; e_2014100596h@uncp.edu.pe (P.Á.-O.); rzquispe@uncp.edu.pe (R.Q.-R.); egarcia@uncp.edu.pe (E.G.-O.); 3 Science Faculty, Universidad Nacional Agraria La Molina, Lima 15024, Peru; erios@lamolina.edu.pe

**Keywords:** bovine ears, bovine blood, bovine cheeks, ruminal content, digestibility, guinea pigs, metabolizable energy, biowaste

## Abstract

**Simple Summary:**

Every year, the consumption of beef cattle increases, as does slaughter waste, including rumen content, blood, and other waste such as ears and cheeks. These can be converted into meals through heat treatment, so it is important to know their nutritional value. In this study, tests were carried out that allowed us to determine the chemical composition, the contribution of available nutrients, and the energy content of this slaughter waste to prepare rations for guinea pigs. After the study, it was verified that flours made from ruminal content, blood, ears, and cheeks are good sources of protein and energy for guinea pigs and are consumed without problems when included as one-fifth of a barley-based diet. This circular economy approach allows this type of waste to be recovered and valued, with the advantage of reducing its polluting power due to inadequate disposal or its disposal into rivers.

**Abstract:**

Biowaste from slaughterhouses can be recovered to benefit food security and reduce contamination potential. More than 3 billion heads of livestock are consumed worldwide, which will increase by 17% by 2028, generating more biowaste, increasing infectious agents, and causing economic losses due to circular economy principles not being applied. This work evaluated the nutritional quality of four types of biowaste from bovine slaughter which were transformed into a meal for guinea pigs (rumen content (RCM), ears (EaM), blood (BM), and cheeks (CM)) according to their chemical composition, digestible components, energy contribution, and voluntary consumption. For the animal model, adult male guinea pigs were arranged in metabolic cages for feces collection without urinary contamination. Nine guinea pigs were used in each digestibility test. First, a direct digestibility test was conducted using a meal of barley as a reference diet (RD), the indigestibility coefficient of which allowed for the estimation of the digestibility of biowaste meals through indirect calculations; for this, diets composed of 80% of the RD and 20% of the corresponding biowaste meals were evaluated. The difference method was suitable for determining the digestibility of beef biowaste using the indigestibility coefficients of the reference diet to calculate the digestibility of ingredients which could not be offered as 100% of the meal but were incorporated as 20%. The digestible protein and metabolizable energy contents of RCM, EaM, BM, and CM were 10.2% and 2853 kcal/kg, 44.5% and 3325 kcal/kg, 70.7% and 2583 kcal/kg, and 80.8% and 3386 kcal/kg, respectively. The CM and BM feeds had the highest contributions of digestible protein due to their higher nitrogen content, and the CM and EaM feeds had the highest ME contents due to their higher fat contents. The biowaste meal consumption in descending order was CM > RCM > EaM > BM, which were consumed without problems. These results are indicative that these components can be part of guinea pigs’ diets, and it is recommended to continue studies into guinea pig growth and fattening diets with different levels of these biowaste meals.

## 1. Introduction

Given population growth and the food needed to sustain it, there is great concern about using protein sources such as soy and fish in feed due to their poor sustainability and high cost, which is forcing researchers to evaluate alternatives for animal nutrition [1].

Raising guinea pigs to consume their meat is important in the Andean countries of Latin America; due to the protein quality and exquisiteness of guinea pig meat, their breeding and consumption have spread exponentially worldwide [2,3,4,5], and guinea pigs are contributing to food security and providing a secure economic income for the population [6]. Official data from Peru report a total of 827,234 ranchers who produced 21,103 tons of guinea pig meat, recording a per capita consumption of 0.66 kg/inhabitant/year in 2017 [7]. More than 71% of guinea pig meat is exported to the United States and, on a smaller scale, it is exported to Japan, Canada, South Korea, Italy, and Aruba [7].

In commercial guinea pig production systems, the diets comprise the main macro ingredients of corn flour; soy cake; whole soy meal; fish meal; wheat sub-products; hominy feed; alfalfa hay; wheat flour, among other vegetable flours; and micro ingredients like vitamin and mineral premixes and synthetic amino acids and additives [8,9,10]. As macro ingredients are increasingly expensive and scarcely available and many are used as food for humans, it is necessary to look for new alternatives, which is why this study evaluates the nutritional quality of various biowaste meals from the slaughter of cattle.

Around the world, the slaughterhouse industry has experienced rapid growth driven by the demand for meat and derivatives, generating a large amount of waste that remains after slaughter, affecting environmental sustainability; this waste could be recovered through waste-to-energy technologies and value-added products [1,11,12], contributing to the environment and public health [11]. It is estimated that global meat production is 220 million tons, and 31% comes from cattle [11]. Between 50 and 54% of the weight of each processed cow is used for meat, and the rest constitutes waste, with much of this being inadequately disposed [13]. Between a third and half of the total weight of an animal is waste that is not used or is partially used [14,15].

In Peru, as in other developing countries, there is limited scientific information on the quantity and quality of slaughterhouse waste, and companies are not willing to provide information and are not aware that waste is dangerous [16]. Their solid waste management practices are inadequate and ineffective [17,18] and generate a notable environmental impact, whose indicator is the high content of organic load in wastewater from slaughterhouses, giving rise to damage to treatment plants and drains such as obstructions and clogging, in addition to allowing the treated water to exceed the maximum permissible values [19].

From the perspective of environmental sustainability and the circular economy, waste from animal slaughter, such as the blood, rumen content, ears, and cheeks of cattle, could be incorporated into animal diets, reducing the environmental risks that are caused by their inappropriate disposal.

Crude protein content (PB) and ethereal extract (EE) have the economic and nutritional potential to be transformed into flours with high nutritional value to formulate balanced diets for minor monogastric species such as guinea pigs; this is an urgent area of research.

It is estimated that for each cow, approximately 4% of live weight (20 L) of blood [20] and 30 kg of rumen content [21] can be collected. It is also indicated that the yields of blood meal and cheek meal are between 10 and 13% of their raw material [22]. The rumen content contains 13.7% ash, 9% CP (Nx5.7), 13% moisture (M), 0.1 EE, 34% crude fiber (CF), 30% nitrogen-free extract (ELN), and 1730 Kcal of energy/kg [14] and can replace up to 40% of soybeans in the diets of some fish without affecting their growth [21].

Blood from slaughterhouses is one of the main animal by-products; it is rich in protein and blood meal (BS) and contains up to 80% CP [23]. Several authors recommend using it in the feed of birds, rabbits, guinea pigs, and ruminants [23,24,25,26,27]. Blood is made up of plasma, cellular fraction, and fibrillar fraction; it contains lipoproteins, non-esterified fatty acids, sugars, and soluble proteins. Therefore, the treatment to produce flour recommends that if it is subjected to high temperatures above 105 °C for more than 2 h, the blood proteins can be burned, and, therefore, its quality and digestibility are very poor. Bovine ears, horns, hooves [28], and cheeks can also be used as food [22].

In this study, rumen content meal (RCM), blood meal (BM), ear meal (EaM), and cheek meal (HM) were prepared and evaluated, and their nutritional value was estimated from three points of view using adult male guinea pigs as an animal model, following a standardized methodology: the proximal chemical composition (PB, EE, FC, ELN, and ash), the apparent digestibility of the proximal components, and the voluntary intake. With these data, the digestible nutrient content and contribution of digestible energy (DE) and metabolizable energy (ME) were determined [26].

## 2. Materials and Methods

### 2.1. Animal Ethics Statement

All experimental protocols in the current study were reviewed and approved by the Specialized Institute of the Faculty of Zootechnics of Universidad Nacional del Centro del Peru. The Animal Research: Reporting of In Vivo Experiments [29] guidelines were followed in all digestibility trials. The sample size allows for enough replications. The animals were arranged individually in metabolic cages that provide comfort and ease of measurement of food consumption and the corresponding feces production. No invasive testing was performed.

### 2.2. Study Location

The digestibility tests were realized in the nutritional evaluation and chemical analysis room in the Animal Nutrition Laboratory of the National University of Central Peru. The collection of biowaste was carried out in slaughterhouses in the central region of Peru, characterized by its tundra climate, with a rugged relief (altitude 3250 to 3350 m.a.s.l.), an approximate rainfall of 1700 mm, and an average annual temperature of 8.7 °C, according to Köppen and Geiger [30].

### 2.3. Experimental Animals

The guinea pigs used in the study came from the Yauris Agricultural Farm of the Universidad Nacional del Centro del Perú. A total of 45 male meat guinea pigs from the Peru line were used that were around 5 months old, of similar weight (822 ± 18 g), healthy, and randomly distributed into 5 groups of 9 animals each, thus guaranteeing that each experimental unit had the same probability of receiving a particular treatment. The number of animals used per experimental group was in line with international recommendations on the care and use of animals for research [31]. Once the experiments were over, all animals were returned to the farm to continue breeding.

### 2.4. Process of Obtaining Bovine Slaughterhouse Biowaste Meals

**Figure 1:**
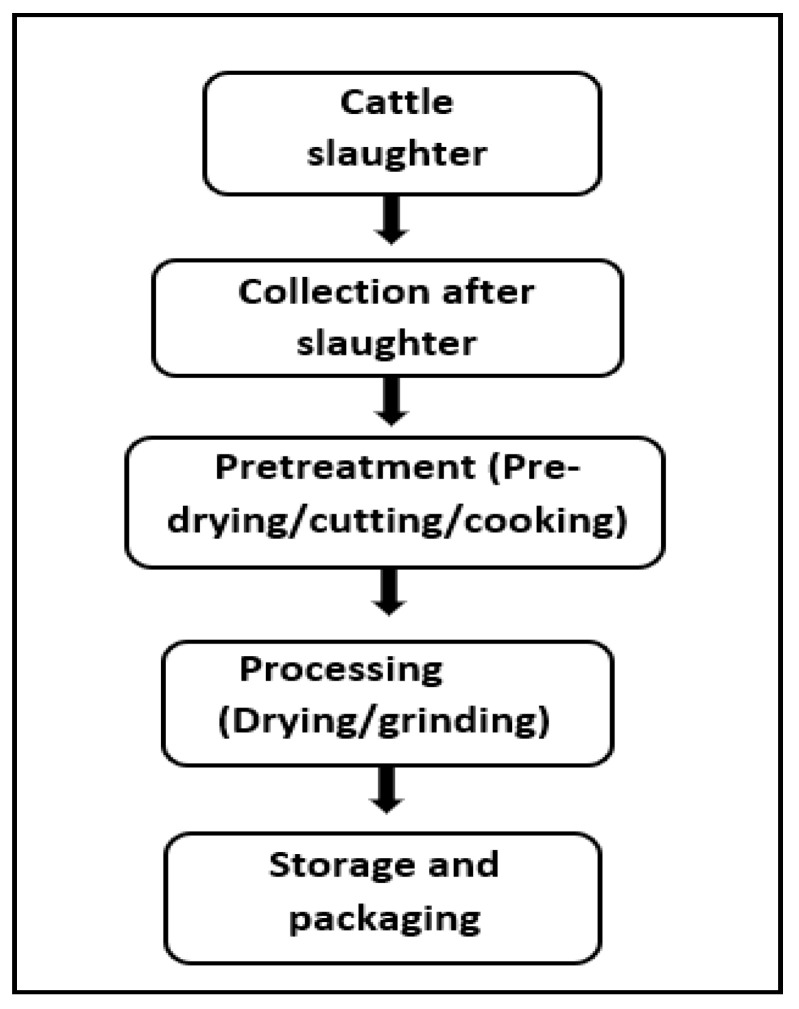
General flow diagram of the process to obtain bovine slaughterhouse biowaste meals.

The different phases for obtaining protein meals from bovine slaughterhouse biowaste generally consider the collection, reception, pretreatment, processing, and storage of the meals (Table 1, Figure 1). The rumen content was dried and ground, while the ear, blood, and cheeks were previously cooked for drying and grinding.

### 2.5. Laboratory Analysis

To carry out proximal analyses, the feces and biowaste meal samples were previously ground in a Wiley Tecnal-TE-648 type micro mill (Tecnal, Piracicaba, Brazil) using a 1 mm sieve. Dry matter (DM), crude protein (CP), crude fiber (CF), ether extract (EE), and ash were analyzed following the corresponding AOAC methods: 934.01, 976.05, 922.06, and 961.14 [32].

### 2.6. Digestibility and Voluntary Consumption Trials of Biowaste Meals

Biowaste meals cannot constitute 100% of the diet. The digestibility coefficients were estimated by difference using the values determined with the reference diet compared to those recorded in a diet that includes a proportion of test food [33]. Assuming that the indigestibility of the reference diet is the same in the diets with 20% beef biowaste meals, with the indigestibility coefficients of the reference diet, mathematical calculations were carried out to determine the digestibility of the biowaste meals [34,35]. 

In each in vivo digestibility test, nine male guinea pigs from the Peru line, around five months old, were used, individually arranged in metabolic cages that allowed feces to be collected without urinary contamination. Barley meal was used as a reference diet (RD), and the indirect method was used to calculate the digestibility coefficients of RCM, BM, EaM, and CM following a previously described methodology [22,35].

The digestibility trials had two stages of seven days each. In the seven pre-experimental days, the guinea pigs adapted to the conditions of the metabolizable cages and the new diets. Taking into account that the microflora and the digestive process responded to the exclusive consumption of forage, in the pre-experimental stage, forage was gradually replaced by the study diets. Of the 300 g of forage that they consumed daily, they were given 50 g less per day, so that at the end of the pre-experimental stage, the guinea pigs consumed only the study diets, which were offered ad libitum from the beginning of the pre-experimental stage (Table 2):

T0: RD diet: 100% barley meal;
T1: RCM diet: 80% RD + 20% RCM;
T2: EaM diet: 80% RD + 20% EaM;
T3: BM diet: 80% RD + 20% BM;
T4: CM diet: 80% RD + 20% CM.

In these studies, diets that covered the nutritional requirements of the guinea pigs were not formulated. We used a feed that can be used 100%, such as barley meal, to determine the digestibility and indigestibility of its proximal components. So, the inputs to be evaluated (biowaste meals) comprised 20% of the diet and were combined with 80% of the reference diet to be able to estimate the digestibility of these feeds by the difference method. The goal was not to compare them but to individually determine their digestible components and their contribution of metabolizable energy to increasing the information in the food tables for guinea pigs.

In the experimental period, feces were weighed, and samples from seven consecutive days were collected in sample bags and stored at −4 °C immediately after collection. Food consumption and the corresponding feces production were measured daily. After the experimental stage, the animals exclusively consumed the test diets and drinking water and were accustomed to the conditions of the metabolic cage. The feces collected from each animal were weighed on a digital scale and dried in a forced convection oven to determine the dry matter content and continue with the chemical analyses. 

Feed was distributed daily at 9:00 a.m., and before offering the day’s ration, the residue from the previous day was weighed to calculate the net daily fresh consumption by subtracting the residue from the quantity of feed offered; this allowed us to calculate the total intake of DM, CP, EE, CF, and FEN, with their corresponding amounts eliminated in the feces, and, using the calculations mentioned in Section 2.7, the apparent digestibility coefficients of the proximal organic components of the biowaste meals. Because the initial and final weights of the animals were taken during the experimental phase, the average daily voluntary consumption was determined as a percentage of live weight and per kilogram of metabolic weight (W^0.75^).

Daily feed residues were not returned to the feeder and were eliminated. Drinking water plus vitamin C was distributed “ad libitum” in clay vessels.

### 2.7. Digestibility Coefficient Calculations and Total Digestible Nutrients

Digestibility is calculated as the amount of a proximal component consumed (I) minus the amount of the same component eliminated in feces (F).

The apparent digestibility coefficients of DM, CP, EE, FB, and ELN were estimated from their corresponding intakes minus the corresponding fractions eliminated in feces, using the equation described by McDonal [36,37]:

$$D=\frac{I-F}{I}\times 100$$

where:

D: percentage of apparent digestibility;

I: amount of nutrients ingested;

F: amount of nutrients excreted.

Total digestible nutrients (TDNs) were estimated using the formula [38]:

$$TDN\left(\%\right)=\frac{\left(\%CP\times Dig\right)+\left(\%EE\times Dig\right)2.25+\left(\%CF\times Dig\right)+\left(\%NFE\times Dig\right)}{100}$$

where:

% CP = biowaste meal crude protein percentage;

% EE = biowaste meal ether extract percentage;

% CF = biowaste meal crude fiber percentage;

% NFE = biowaste meal nitrogen free extract percentage;

Dig = percent digestibility of the respective nutrient.

The contents of digestible energy (DE) and metabolizable energy (ME) in Kcal/kg were estimated by multiplying the percentage of TDNs by 44.09 and the DE by 0.82, respectively [39,40].

### 2.8. Statistical Analysis

The processing of the data obtained for each variable studied was through a descriptive analysis to summarize the data. Comparisons between the digestibility coefficients and voluntary consumption of the evaluated foods were carried out through analysis of variance in SPSS V23 with 0.05 confidence. For multiple comparisons, Duncan’s test was used, which allows for a comparison of the means between the parameters obtained between different biowaste meals.

## 3. Results

### 3.1. Chemical Composition of Biowaste Meals from Cattle Slaughter

The analysis of the proximal chemical composition of the biowaste meals from a cattle slaughter is shown in Table 3. The ear, blood, and cheek meal do not have CF or NFE, while the ruminal content meal has CF.

### 3.2. Apparent Digestibility Coefficients of Slaughter Biowaste Meals

The digestibility coefficients of slaughter bovine biowaste meals are shown in Table 4.

### 3.3. Digestible Components of Slaughter Biowaste Meals and Energy Contribution

All biowaste meals have high contents of total digestible nutrients, which determine the main contributions of digestible and metabolizable energy to be used in the formulation of diets for the different physiological phases of guinea pigs (Table 5).

### 3.4. Live Weights of Experimental Animals

At the beginning of the digestibility experiments, the live weights of the guinea pigs had similar averages by treatment (*p* > 0.05). When evaluating the weight gains during the 7 experimental days, and the daily weight gains per animal, significant differences were recorded (*p* < 0.05). The weight gains of animals that had access to the diet with rumen content were lower than those that consumed the diets with ear, blood, and cheek meals (Table 6).

### 3.5. Voluntary Consumption of Bovine Slaughter Biowaste Meals

Dry matter consumption of biowaste meals, as a percentage of live weight, was between 0.45 and 0.79%, being consumed by guinea pigs without a problem when included in 20% of the diet (Table 7).

## 4. Discussion

To our knowledge, this study is the first to evaluate the nutritional quality of ear and cheek meal from a cattle slaughter, with some studies on blood meal and rumen content in species other than guinea pigs.

### 4.1. Approximate Composition of Bovine Slaughter Biowaste Meals

Blood, ear, and cheek meal have a high protein and energy content and can be used as protein sources in the diet of guinea pigs, taking into account the variability in the proximal chemical composition reported in other studies, such as the case of ruminal content and blood meal (Table 8), with no information found for ear meal and cheek meal.

### 4.2. Apparent Digestibility of Bovine Slaughter Biowaste Meals

Our results demonstrate that the digestibility of ruminal content meal is higher than some flours of plant origin, such as soybean and quinoa (62.7 and 64.1% of the total digestibility) [53]. In the case of the DM ingested from the rumen content, 83% was absorbed, and it contains highly digestible microbial protein. The protein digestibility of the ruminal content was above 67%, and that of EE, CF, and NFE was above 77%. This trend of results is indicative that this wasted by-product can be used well in guinea pig diets, with the advantage of reducing environmental pollution due to its inadequate disposal.

The DM digestibility values of ear meal were higher than those of poultry meal (73%), pork meal (69%), feather meal (62%), and even blood meal (57%), while the digestibility of crude protein was close to poultry meal (78–80%) and pork meal (76%) and higher than blood meal and feather meal (65–67%) [54].

It is reported that the digestibility of protein-rich ingredients is between 75 and 95% [26]. In this study, the digestibility of dry matter and protein of the ruminal content meal, composed of ingredients of plant origin, was even higher than that reported in Cachama fish [55], a result that, although it does not directly explain our specific findings, is interesting to consider, because fish have a simple stomach, with its own morphological and physiological characteristics and dietary adaptations, with rapid transit of food through the intestine and little microbial fermentation [56,57]. Guinea pigs use fiber better and have more fermentative activity; they use the ruminal content better than Cachama fish, demonstrating its high digestibility for guinea pigs.

In general, researchers agree that ingredients of animal origin have higher digestibility coefficients than vegetables [53], as observed in this study concerning ruminal content composed mainly of forage.

The blood meal crude protein digestibility was above 97%, which allows it to be valued as a good protein input for guinea pigs, which are not very demanding in amino acid quality, as is the case with aquaculture species, chickens, and pigs, which mainly require the important contribution of limiting amino acids such as methionine and lysine.

In this study, the cheek meal protein digestibility was above 96%, and that of the ether extract was above 82%, which means they are good inputs for guinea pig diets.

### 4.3. Total Digestible Nutrients of Bovine Slaughter Biowaste Meals

The TDN concentration of the ruminal content meal for guinea pigs was close to 79%, a value that can be validly used for the formulation of rations in this animal species. This TDN content is like that reported for blood meal (81%) and fish meal (76%) but lower than that reported in fish viscera meal (97.7%) [26], which agrees with other results in which it was observed that the higher the fiber content, the lower the energy content [58].

The TDN content of bovine ear meal for guinea pigs was close to 92%, a value higher than that recorded in blood meal (81%) and fish meal (76%) but lower than the TDN content of fish viscera meal (98%), as reported in a previous study [26]. This input could be the main protein carrier in guinea pig diets.

The TDN content of bovine blood meal was higher than 71%, and it has a high protein value for guinea pigs, a species that is becoming increasingly important in the national and international markets, which is important to evaluate when replacing other grains that are less available and expensive. The TDN content of blood meal was slightly lower than that reported for fish meal (76%) and fish viscera meal (98%) [26].

The results of this study demonstrate that meals produced from cattle slaughter waste not only have a high protein content but also a high energy content in terms of TDNs and can be incorporated into the classification of protein energy ingredients for feeding guinea pigs.

The biowaste meals’ digestible energy contents were between 3480 and 4129 kcal/kg, being higher in the order RCM < BM < EaM < CM; a trend like that registers the contributions of metabolizable energy, where CM had the highest level of metabolizable energy for guinea pigs. These results provide additional information regarding the chemical composition of foods for meat guinea pigs [26].

### 4.4. Live Weight and Weight Gain per Guinea Pig per Treatment

The average live weight of the guinea pigs at the beginning of the experimental phase was similar in all treatments (*p* > 0.05). After the digestibility test, daily weight gains were between 4.08 and 7.02 g, with similar values in the diets containing EaM, BM, and CM (Table 5). The lowest weight gains were recorded in the diet with RCM, an expected result due to its higher fiber content and low protein content compared to the other protein residues.

Diets with 20% EaM, BM, or CM had 22.13%, 24.08%, and 26.44% CP, while the diet with 20% ruminal content had only 12.6% CP; we observed that diets with a higher level of crude protein significantly improved weight gains (*p* < 0.05). These results demonstrate that meal waste from cattle slaughter can be used in the formulation of rations for guinea pigs, which was also reported in other studies [48,55,59].

Some studies demonstrate the advantages of using blood meal and ruminal content in feeding guinea pigs, but there are still no reports that have used ear meal and cheek meal, which gives originality to this work. 

A study in Cotopaxi, Ecuador, which evaluated three levels of blood meal—T1 (2%), T2 (4%), and T3 (6%)—reported similar weight gains, only in carcass yield for T3, and the authors suggested using a diet with 6% BM [59]. 

The use of blood meal and forage in the growth and fattening stages has advantages due to their high digestibility.

Another study [51] evaluated the inclusion of 0, 4, 8, and 12% bovine blood meal in guinea pig diets, and the 12% level yielded improved nutritional and economic efficiency compared to the control group without blood meal.

When evaluating productive behavior in fattening guinea pigs using a 5, 10, and 15% rumen content, they reported that a 15% rumen content use allowed for the best weight gains and greater feed efficiency, and, in general, the guinea pigs consumed the total mixed feed provided and left no residue. In the bromatological analysis, the rumen content presented 13.7% ash, 9% (Nx5.7) protein, 12.6% moisture, 0.1% fat, 34.1% crude fiber, 30.5% total carbohydrates, and 1730 Kcal/kg [45].

### 4.5. Voluntary Consumption of Biowaste Meals from Bovine Slaughter

Knowledge of the palatability of feeds has nutritional importance and is correlated with their nutritional value [60]. For this reason, it is important to evaluate the voluntary consumption of the different inputs incorporated into animal diets [61].

In this study, the daily average intake of biowaste meals was determined, and the percentage of the live weight was between 0.45 and 0.79%, with the level of consumption from less to more being BM < EaM < RCM < CM. 

A similar trend was recorded concerning the consumption per kg of metabolic weight (10 to 40 g), in the following order: BM < EaM < RCM < CM. 

These results are like those reported in other studies [26], and what is important is the acceptance of these feeds by the guinea pigs, which stimulates weight gain and performance [62].

Consumption depends on the quality of the feeds and the energy content of diets, and it can increase significantly when diets are low in energy compared to diets with higher energy concentrations [9].

## 5. Conclusions

The difference method was suitable for determining the digestibility of beef biowaste using the indigestibility coefficients of the reference diet for calculating the digestibility of ingredients that cannot be offered at 100% but incorporated at a level of 20%.

Meals made with bovine ruminal content, blood, ears, and cheeks constitute a source of nutrients with good protein digestibility and contribution to metabolizable energy, and they were consumed without problem by the guinea pigs. Therefore, they could be used in their diet, and the ecosystems surrounding bovine slaughter centers would be improved, reducing environmental pollution and contributing to the circular economy and public health.

The digestible protein and metabolizable energy contents of the ruminal content, ear meal, blood meal, and cheek meal were 10.15% and 2853 kcal/kg, 44.54% and 3325 kcal/kg, 70.68% and 2583 kcal/kg, and 80.84% and 3386 kcal/kg.

## Figures and Tables

**Table 1:** Production phases of bovine slaughterhouse biowaste meals.

Biowaste	Collection	Reception	Pretreatment	Processing	Storage and Packaging
Ruminal content meal (RCM)	After slaughter and removal of the rumen reticulum from the digestive tract	Polyethylene buckets Drained on a 0.1 mm sieve	Pre-drying under shade (3 days), with dough movements 2 times/day	Drying by forced convection (Binder: FED20) 80 °C/4 h. Grinding (2 mm screen)	Polyethylene bags
Ear meal (EaM)	Cutting, washing, and rinsing the ears	Hair removal. Immerse in water at 70 °C/2 min. Peeling with blade	Cooking 90 °C/10 min. Chopped (2 cm^2^)	Drying in a forced convection oven at 80 °C/8 h. Grinding (2 mm screen)	Polyethylene bags
Blood meal (BM)	During the benefit after the jugular cut	Polyethylene buckets	Coagulation by cooking in a steel container (90 °C/10 min) with constant stirring and cutting (2 cm^2^)	Drying in a forced convection oven at 80 °C/8 h. Grinding (2 mm screen)	Polyethylene bags
Cheek meal (CM)	Cutting, lacquering, and rinsing cheeks	Hair removal. Immerse in water at 70 °C/2 min.	Cooking 90 °C/10 min	Cut into pieces (2 cm^2^) and dried in a forced convection oven at 80 °C/8 h. Grinding (2 mm screen)	Polyethylene bags

Source: Own elaboration.

**Table 2:** Formulas and chemical composition of the evaluated rations.

	Reference Diet (RD)	RD + 80% RCM	RD + 80% EaM	RD + 80% BM	RD + 80% CM
T0	100	80	80	80	80
T1	0	20	0	0	0
T2	0	0	20	0	0
T3	0	0	0	20	0
T4	0	0	0	0	20
Total, %	100	100	100	100	100
DM, %	91	89.80	90.58	91.20	91.15
CP, %	12	12.60	22.13	24.08	26.44
EE, %	4	4.30	7.68	3.30	4.58
CF, %	5.8	12.24	4.64	4.64	4.64
ME, kcal/kg	2884	2877.80	2972.20	2823.80	2984.40

The CF contents of RD + 80% EaM, RD + 80% BM, and RD + 80% CM give the same value (4.64%) because EaM, BM, and CM do not contain CF, so the CF content of these three rations corresponds only to the DR.

**Table 3:** Proximate analysis of slaughter bovine biowaste meals.

Biowaste Meals	DM (%)	CP (%)	EE (%)	CF (%)	NFE (%)	Ash (%)
RCM	85.00 ± 0.33	15.00 ± 0.30	5.50 ± 0.30	38.00 ± 1.37	30.50 ± 1.84	11.00 ± 1.07
EaM	88.91 ± 1.32	62.64 ± 0.61	22.4 ± 0.52	†	†	14.86 ± 0.13
BM	92.00 ± 0.30	72.40 ± 1.19	0.50 ± 0.06	†	†	4.00 ± 0.16
CM	91.76 ± 0.02	84.19 ± 0.02	6.91 ± 0.03	†	†	8.90 ± 0.01

RCM: ruminal content meal; EaM: ear meal; BM: blood meal; CM: cheek meal; DM: dry matter; CP: crude protein; EE: ethereal extract; CF: crude fiber; NFE: nitrogen-free extract. †: absence of CF and NFE.

**Table 4:** Apparent digestibility coefficients of slaughter bovine biowaste meals.

Biowaste Meals	DM (%)	CP (%)	EE (%)	CF (%)	NFE (%)
RCM	83.05 ± 5.51 ^a^	67.65 ± 4.69 ^b^	82.76 ± 11.35 ^b^	91.52 ± 4.73	77.91 ± 9.21
EaM	88.28 ± 2.38 ^a^	71.10 ± 7.95 ^b^	94.11 ± 3.02 ^a^	-	-
BM	88.53 ± 3.20 ^a^	97.63 ± 0.74 ^a^	65.25 ± 20.77 ^c^	-	-
CM	76.34 ± 1.53 ^b^	96.03 ± 0.39 ^a^	82.37 ± 1.28 ^b^	-	-

^a,b,c^ Values per component with different letters vary statistically (*p* < 0.05). RCM: ruminal content meal; EaM: ear meal; BM: blood meal; CM: cheek meal; DM: dry matter; CP: crude protein; EE: ethereal extract; CF: crude fiber; NFE: nitrogen-free extract.

**Table 5:** Digestible components and energy contribution of slaughter bovine biowaste meals.

Biowaste Meals	MS (%)	PB (%)	EE (%)	CF (%)	ELN (%)	NDT (%)	ED (Kcal/kg)	ME (Kcal/kg)
RCM	70.60 ± 4.69	10.15 ± 0.70 ^d^	4.55 ± 0.62	34.78 ± 1.80	23.76 ± 2.81	78.93 ± 5.36	3480 ± 236	2853 ± 194 ^b^
EaM	78.49 ± 2.12	44.54 ± 4.98 ^c^	21.08 ± 0.68	-	-	91.97 ± 6.02	4055 ± 265	3325 ± 217 ^a^
BM	81.45 ± 2.95	70.68 ± 0.54 ^b^	0.35 ± 0.09	-	-	71.46 ± 0.56	3151 ± 24.8	2583 ± 20 ^c^
CM	70.05 ± 1.40	80.84 ± 0.33 ^a^	5.69 ± 0.09	-	-	93.65 ± 0.53	4129 ± 23.26	3386 ± 19 ^a^

^a,b,c,d^ Average values per type of residue with different letters vary statistically (*p* < 0.05). RCM: ruminal content meal; EaM: ear meal; BM: blood meal; CM: cheek meal; DM: dry matter; CP: crude protein; EE: ethereal extract; CF: crude fiber; NFE: nitrogen-free extract; TDNs: total digestible nutrients; DE: digestible energy; ME: metabolizable energy.

**Table 6:** Initial and final weights and total and daily weight gains of the guinea pigs during the experimental phase (7 days).

Parameter	RCM	EaM	BM	CM
Initial weight means, g	807.78 ± 68.75 ^a^	834.44 ± 53.35 ^a^	840.33 ± 67.17 ^a^	806.11 ± 58.28 ^a^
Final weight means, g	836.33 ± 65.82 ^a^	878.11 ± 53.17 ^a^	888.44 ± 63.6 ^a^	855.22 ± 55.51 ^a^
Total weight gain means, g	28.56 ± 5.48 ^b^	43.67 ± 8.14 ^a^	48.11 ± 8.93 ^a^	49.11 ± 7.22 ^a^
Daily weight gain means, g	4.08 ± 0.78 ^b^	6.24 ± 1.16 ^a^	6.87 ± 1.27 ^a^	7.02 ± 1.03 ^a^

^a,b^ Average values per type of residue with different letters vary statistically (*p* < 0.05).

**Table 7:** Voluntary dry matter consumption of biowaste meals as a percentage of live weight (LW) and in grams per kilogram of metabolic weight (Kg W^0.75^) in guinea pigs.

Biowaste Meals *	Voluntary Dry Matter Consumption	
	Live Weight Percentage	g/Kg W^0.75^
RCM	0.65 ± 0.23 ^b^	12.41 ± 2.03 ^b^
EaM	0.45 ± 0.17 ^b^	10.15 ± 1.76 ^b^
BM	0.58 ± 0.22 ^b^	11.55 ± 2.19 ^b^
CM	0.79 ± 0.07 ^a^	39.97 ± 2.75 ^a^

^a,b,^ Values per biowaste meals with different letters vary statistically (*p* < 0.05). RCM: ruminal content meal; EaM: ear meal; BM: blood meal; CM: cheek meal. * In the digestibility test diets, biowaste flours entered 20%. They all had 80% barley flour. The consumption calculations correspond exclusively to biowaste flours using the difference method.

**Table 8:** Research related to the present study.

Rumen Content Meal:		Humidity	Crude Protein	Crude Fiber	Ethereal Extract	Ash	NFE
Our study	Ruminal content	15.00	15.00	38.00	5.50	11.00	30.50
[41] 2015	Ruminal content	7.36	18.26	24.99	3.60	14.47	ud
[42] 2011	Fermented bovine blood and rumen digesta	7.20	29.86	21.90	23.50	7.40	12.14
[43] 2010	Sun-dried ruminal content	7.95	13.56	31.90	0.75	16.20	25.70
[44] 2008	Sun-dried ruminal content	71.15	12.85	9.45	3.35	8.36	37.14
[45] 2016	Ruminal content	87.40	9.00	34.1	0.098	13.70	30.50
[46] 2017	Ruminal content	8.80	6.77	21.99	0.00	17.11	ud
[47] 2018	Ruminal content	12.00	13.00	27.00	2.00	ud	ud
[48] 2014	Ruminal content	12.60	9.00	27.00	0.10	13.70	30.50
[49] 2006	Fresh ruminal content	87.85	ud	ud	ud	14.40	ud
Blood meal:							
Our study	Blood meal	8.00	72.40	0.00	0.50	4.00	0.00
[50] 2018	Blood meal		92.50	0.00	7.50		0.00
[41] 2015	Blood meal	10.13	84.87	0.38	0.52	4.66	
[51] 2017	Blood meal		75.76		1.03	4.87	
[51] 2017	Blood meal		72.14		1.96	7.38	
[52] 2013	Blood meal	4.40	72.00		<1.00		
[26] 2021	Blood meal	11.85	92.25	0.00	0.56		7.19
Ear meal:							
Our study	Ear meal	11.09	62.64	0.00	22.40	14.86	0.00
There are no reports							
Cheek meal:							
Our study	Cheek meal	8.24	84.19	0.00	6.91	8.90	0.00

ud: undetermined.

## Data Availability

The study data may be made available when interested parties request them from the corresponding author.
